# Real-Time Sensing of Enteropathogenic *E. coli*-Induced Effects on Epithelial Host Cell Height, Cell-Substrate Interactions, and Endocytic Processes by Infrared Surface Plasmon Spectroscopy

**DOI:** 10.1371/journal.pone.0078431

**Published:** 2013-10-23

**Authors:** Victor Yashunsky, Leorah Kharilker, Efrat Zlotkin-Rivkin, David Rund, Naomi Melamed-Book, Eitan Erez Zahavi, Eran Perlson, Silvana Mercone, Michael Golosovsky, Dan Davidov, Benjamin Aroeti

**Affiliations:** 1 The Racah Institute of Physics, the Hebrew University of Jerusalem, Jerusalem, Israel; 2 Department of Cell and Developmental Biology, and the Bioimaging Unit, The Alexander Silberman Institute of Life Sciences, The Hebrew University of Jerusalem, Jerusalem, Israel; 3 The Institute of Biochemistry, Food Science and Nutrition, Robert H. Smith Faculty of Agriculture, Food and Environment, The Hebrew University of Jerusalem, Rehovot, Israel; 4 Department of Physiology and Pharmacology, Sackler Faculty of Medicine, Tel-Aviv University, Ramat Aviv, Tel Aviv, Israel; 5 Université Paris 13, Sorbonne Paris Cité, LSPM–(UPR 3407) CNRS, Clément, Villetaneuse, France; 6 Confocal Unit, The Alexander Silberman Institute of Life Sciences, The Hebrew University of Jerusalem, Givat Ram, Jerusalem, Israel; University of Florida, College of Dentistry & The Emerging Pathogens Institute, United States of America

## Abstract

Enteropathogenic *Escherichia coli* (EPEC) is an important, generally non-invasive, bacterial pathogen that causes diarrhea in humans. The microbe infects mainly the enterocytes of the small intestine. Here we have applied our newly developed infrared surface plasmon resonance (IR-SPR) spectroscopy approach to study how EPEC infection affects epithelial host cells. The IR-SPR experiments showed that EPEC infection results in a robust reduction in the refractive index of the infected cells. Assisted by confocal and total internal reflection microscopy, we discovered that the microbe dilates the intercellular gaps and induces the appearance of fluid-phase-filled pinocytic vesicles in the lower basolateral regions of the host epithelial cells. Partial cell detachment from the underlying substratum was also observed. Finally, the waveguide mode observed by our IR-SPR analyses showed that EPEC infection decreases the host cell's height to some extent. Together, these observations reveal novel impacts of the pathogen on the host cell architecture and endocytic functions. We suggest that these changes may induce the infiltration of a watery environment into the host cell, and potentially lead to failure of the epithelium barrier functions. Our findings also indicate the great potential of the label-free IR-SPR approach to study the dynamics of host-pathogen interactions with high spatiotemporal sensitivity.

## Introduction

Enteropathogenic *Escherichia coli* (EPEC) infection is a major cause of infant diarrhea in the developing world [1]. The microbe colonizes the apical surface of the small intestine’s epithelial cells, where it forms characteristic attaching and effacing (A/E) lesions. EPEC utilizes a type-III secretion system (T3SS) to introduce bacterial effector proteins into its host epithelial cells. Several effectors have been implicated in brush border remodeling and the induction of the A/E effects, which significantly contribute to EPEC pathogenesis (recently reviewed in 2). These include effectors that promote local effacement of microvilli, intimate bacterial attachment to the host, and the induction of F-actin-rich protrusions beneath the adhering bacteria, often termed actin-rich pedestals [3]. 

 Type-III-secreted virulent effectors can also disrupt the integrity of the epithelial cell monolayer. For instance, previous studies have reported that several effectors (e.g., EspG, EspF, Map, and NleA) are involved in disrupting the epithelial tight junctions’ (TJs) structure and barrier functions [4–9], when other intercellular junctions, such as desmosomes, remain unperturbed [10,11]. Focal adhesions are affected by EPEC infection in a T3SS-dependent manner, but specific effector(s) that mediate this effect have not yet been identified [12]. A conceivable hypothesis is that the effects that EPEC infection has on intercellular junctions, focal adhesions, and the cytoskeleton would impact the overall epithelial host cell structure and cell monolayer integrity and organization. However, despite the importance of these effects, little research has been conducted to investigate them. 

We have recently developed infrared surface plasmon resonance (IR-SPR) spectroscopy as a novel biophysical tool for studying living cells [13]. For example, we have utilized the surface plasmon and waveguide (TM) modes in the infrared wavelength range to study the epithelial cell monolayer morphology [14,15]. We have also used the IR-SPR method to study the kinetics of endocytic processes with high temporal resolution [16]. Here, we applied this technique to study whether EPEC infection affects the epithelial host cell structure. Importantly, we found that EPEC infection results in a significant blue-shift of the surface plasmon (SPR) and the waveguide (TM) resonances. This implies that during infection the microbe reduces the host cell's refractive index and shortens its height. Using independent bioimaging analyses, we showed that infection with EPEC induces partial cell detachment from the underlying substratum and elevates the levels of (macro)pinocytic-like vesicles in the lower basolateral parts of the host cells. It was reasonable to assume that these effects induced a watery environment to infiltrate into the epithelial hosts, particularly at the cell-substrate interface, which contributes to a decreased refractive index. Our observations hence show, for the first time, that the IR-SPR approach can report on the dynamics of host- pathogen interactions in real-time and in a label-free manner.

## Materials and Methods

### The basics of surface-plasmon spectroscopy

The surface plasmon resonance at the metal-dielectric interface is excited when the *p*-polarized collimated optical beam is reflected from this interface at a certain angle. The resonance conditions correspond to minimal reflectivity in the regime of the attenuated total internal reflection. The resonance wavenumber yields the effective refractive index of the dielectric analyte in the vicinity of the metal. Conceptually, the surface plasmon approach is similar to the total internal reflection (TIRF) technique, since both are based on evanescent wave probing. Although the effective probing distance of the visible surface plasmon wave is limited to ~0.1 µm (which is roughly the same as for standard quantitative total internal reflection microscopy), the probing depth of the surface plasmon wave in the infrared range amounts to several microns, making it advantageous for deep probing into the cell monolayer [13].

To obtain quantitative information using the surface plasmon technique, we express the resonant condition as follows:

$$\nu_{sp}\approx \frac{n_{eff}k_{0}\sin\theta }{2\pi n_{prism}{\left(\varepsilon_{m}^{-1}+n_{eff}^{-2}\right)}^{\frac{1}{2}}}$$ (1)

where ν=1/λ is the surface plasmon resonance wavenumber, *k*_*0*_ is the incident wave vector, Θ is the incident angle, *ε*_*m*_ is the real part of the dielectric permittivity of metal, and *n*_*eff*_ is the effective refractive index of the dielectric (analyte) in the volume probed by a surface plasmon. For living epithelial cells on substrate, *n*_*eff*_= *n*_*medium*_+*f*(*n*_*cell*_-*n*_*medium*_), where *n*_*medium*_, *n*_*cell*_ are the refractive indices of the extracellular medium and the cell layer, respectively, and *f* is the cell coverage. Equation 1 shows that the surface plasmon wavenumber, ν_sp_, is directly related to the *n*_*eff*_ value, which is strongly dependent on the cell layer morphology [17–20]. Therefore, by monitoring the ν_sp_ value, one can measure the cell refractive index, *n*_*cell*_, and the cell coverage, *f*.

In particular, the effective refractive index of a tight and continuous cell monolayer is *n*_*eff*_=*n*_*medium*_+ *Δn*, where *Δn* ^≈^0.03 (at ν~4000 cm^-1^) [21]. The larger refractive index of the cells is attributed to the presence of organic constituents (e.g., nucleic acids and proteins) that have higher refractive indices than water does (the extracellular medium consists predominantly of water, whereas the cell consists of ~ 30% organic constituents). Thus, disruption of the cell monolayer is expected to induce a watery extracellular medium to infiltrate into the cell layer, and induce a decreased effective refractive index of that layer. 

### Leaky waveguide mode in epithelial cell monolayers

An epithelial cell monolayer cultured on an Au-coated ZnS prism is, in fact, a dielectric multilayer with progressively decreasing refractive indices (i.e. ZnS substrate/ cell monolayer/ cell medium). Since cell monolayer thickness (~10 µm) is on the order of several mid-IR wavelengths, it allows excitation of leaky waveguide modes [22] that propagate within the cell monolayer [23]. Such waveguide modes enable direct monitoring of the average cell height and the integrity of an entire cell monolayer, as demonstrated by us recently [23,24]. Waveguide modes, which appear as additional short-wave satellites of the surface plasmon resonance, can be detected by the same optical setup used for surface plasmon spectroscopy. Their resonance wavenumber (ν_TM_) is proportional to the average height of the cells in an intact cell monolayer. Given the optical parameters of the cells [21,25], one can obtain the average cell height from the ν_TM_ value [23].

### Cell culturing on an Au-coated ZnS prism

 Madin Darby canine kidney (MDCK) cells (type-II) were routinely cultured in growth Earl's MEM medium supplemented with 5% Fetal Bovine Serum, 1% non-essential amino acid solution, and 1% antibiotic solution (Biological Industries; Beit Haemek, Israel), as described in ref [23]. A week before the experiment, the confluent MDCK cell monolayer was trypsinized using 0.25% Trypsin 0.02% EDTA solution (Biological Industries; Beit Haemek, Israel). Cells were suspended in 10 ml growth medium, and 1ml of the cell suspension (10^6^cells) was placed on the surface of a Au-coated ZnS prism mounted on a polycarbonate base, as described previously [23]. Cells were allowed to attach to the Au-coated surface for 20 minutes (5% CO_2_, 37 °C, 90% humidity), and then an additional 9 ml of growth medium was added gently to the attached cells. Growth medium was replaced daily. Cells were incubated in a CO_2_ incubator for 6-7 days until a tight epithelial monolayer was achieved. Notably, previous studies have shown that MDCK seeded at an high confluence on glass coverslips, and cultured for at least 4-5 days, form a fully polarized cell monolayer [26].

### Bacterial strains, bacterial activation, and host cell infection

Table **S1** lists the bacterial strains used in this study. Bacteria were grown in Luria Broth (LB) medium and their activation was performed as described [27], except that the activation medium (Minimal Essential Medium, MEM) contained 20 mM Hepes (pH 7.4). Cells were typically infected at a multiplicity of infection (MOI) of 10, and in some cases at an MOI of 5. 

### Infrared surface plasmon monitoring of cell infection

The ZnS prism with MDCK cell culture was attached to a pre-warmed (37°C) flow chamber, filled with pre-warmed Earl’s MEM supplemented with 20mM HEPES (pH=7.0) and was installed within the IR-SPR setup for collimated infrared reflection spectroscopy (see ref [14]). We measured *p*-polarized reflectivity at an oblique angle, Θ=22.5±0.5°, corresponding to the excitation of the surface plasmon resonance at the prism-cell interface at ν=4000 cm^-1^ (λ=2.5 μm). The *s*-polarized reflectivity spectrum at the same incident angle served as a background. The spectra were collected using a Bruker FTIR spectrometer and OPUS software (Bruker Optik GmbH, Ettlingen, Germany). The measurements were performed at 8 cm^−1^ resolution and represented an average over 8 scans, which lasted for 25 sec with a repetition rate of 70 sec. 

The measurement protocol was as follows. We continually measured the IR reflectivity spectra before and while injecting bacteria. The pre-activated bacteria suspended in MEM/Hepes/5% FCS were continuously injected into the flow chamber at a rate of 50 µl/min for 30 min. Following bacteria injection, plain MEM/Hepes/FCS medium was introduced into the chamber at a 0.5 µl/min flow rate for the rest of the measurement time. At the end of each experiment, cells were removed from the Au substrate by introducing Trypsin/EDTA solution into the flow chamber for 30 min. 

### Data processing and elucidating the cell layer refractive index

We measured infrared reflectivity spectra from the cell-prism assembly at a given incident angle, and we found the resonance wavenumber of the surface plasmon resonance. Although the effective refractive index of the analyte (cell layer) can be roughly estimated from Eq. **1**, we used a rigorous procedure, namely, we fitted each spectrum by the model based on Fresnel equations for reflection from the four-layer assembly: a ZnS prism, a 20 nm Au layer, a cell monolayer, and the aqueous medium. This was done using MATLAB software (MATLAB 7.10, The MathWorks, Inc. 1994-2013), based on the algorithm described in ref [28]. The refractive indices of ZnS, Au film, and water, which represented the extracellular medium, were taken from Palik [29]. The difference between refractive indices of the bulk Au and the thin Au film was taken into account, as described in Zilbershtein et al. [21]. The only fitting parameter was the effective refractive index of the cell monolayer. Since the surface plasmon at ν=4000 cm^-1^ is characterized by the ~2 µm penetration depth into the cell layer and the ~50 µm propagation length along the layer [13,24], the effective refractive index measured by SPR is an average over dozens of cells at their basal plane. The effective refractive index is also a weighted average of the refractive indices of the organic substances in the cells, the intracellular water, and the refractive index of the extracellular medium; thus, it is sensitive to disruption of the cell layer and to changes in the water contents of the cells, as a result of the bacterial infection.

### Confocal live-cell imaging

An MDCK cell clone stably expressing LifeAct-GFP (Ibidi, Martinsried, Germany; [30]) was generated using Lipofectamine™2000 transfection reagent (Invitrogen Life Technologies; Carlsbad, CA). Cells were platted on an 8-well tissue culture-treated µ-slide (Ibidi, 80826) at a 1×10^4^ cells/cm^2^ density, and grown for six days in growth medium until a tight cell monolayer was formed. Immediately before the experiment, cells were washed with growth medium. The LifeAct-GFP (Ex: 488nm Em: 505-525) expressing cells were then infected with pre-activated bacteria in MEM supplemented with 5% FCS and 1µM sulforhodamine B (SRB, Biotium, Hayward, CA; Ex: 561nm, Em: 575-625 nm) in the confocal set-up. Cells were imaged by a FV-1000 confocal microscope (Olympus, Tokio, Japan) consisting of an IX81 inverted microscope equipped with a humidified 37 °C incubator supplemented with 5% CO_2_. We captured in 10-minute intervals a z-stack of 25-30 optical sections using a 60 X/1.35NA oil immersion objective. Since the thickness of each section was 0.5 µm, the ~30 sections spanned the entire cell height. Our confocal analyses have shown that the average cell height of the MDCK cells cultured on glass coverslips and grown to superconfluence was similar to that measured for polarized MDCK cells cultured on filters (i.e., 8-15 μm, see ref [31]). A similar average cell height was observed upon infection with EPEC-*wt*, or with other *E. coli* strains. 

### Total internal reflection (TIRF) microscopy

MDCK cells were cultured on glass bottom dishes and transiently transfected with pVSVG-YFP (kindly provided by Dr. Koret Hirschberg, Tel-Aviv University) using the Fugene-6 transfection reagent (Promega, WI, USA). Live cell imaging experiments were carried out a couple of days following transfection on fully confluent cell monolayers. EPEC strains were pre-activated in DMEM prior to infection as detailed above. MDCK cell growth medium was replaced by MEM supplemented with 5% FCS, L-glutamine, and 25mM HEPES, pH 7.4 (without antibiotics). Imaging was carried out on a Nikon Ti Eclipse microscope equipped with a motorized TIRF illuminator (Nikon, Japan) controlled by Andor IQ2 software (Belfast, UK) and the heating chamber was maintained at 37°C. Fluorescence was excited by an Andor 488nm 50mW DPSS laser. The excitation angle was set to give minimal penetration depth while maintaining discernible and homogenous signals, and it remained unchanged between trials. Images were acquired by a Neo sCMOS camera (Andor, Belfast UK). Time lapse images in the TIRF and Bright-field (BF) channels of VSVG-YFP-expressing cells were taken at 1-minute intervals for at least 20 minutes prior to EPEC infection and up to 120 minutes after infection. Images represent at least three independent experiments. The TIRF intensity of VSVG-YFP of the whole cell basal area was measured and corrected for photobleaching as follows: TIRF intensity of VSVG-YFP from the whole cell area was averaged over 3-4 untreated cells, which were imaged under identical conditions. The time dependence of the TIRF intensity was fitted by a logarithmic function and used to correct for photobleaching data obtained for the EPEC-infected cells.

## Results

### EPEC-wt infection decreases the refractive index and the average cell height of the host MDCK cell monolayer

**Figure 1:**
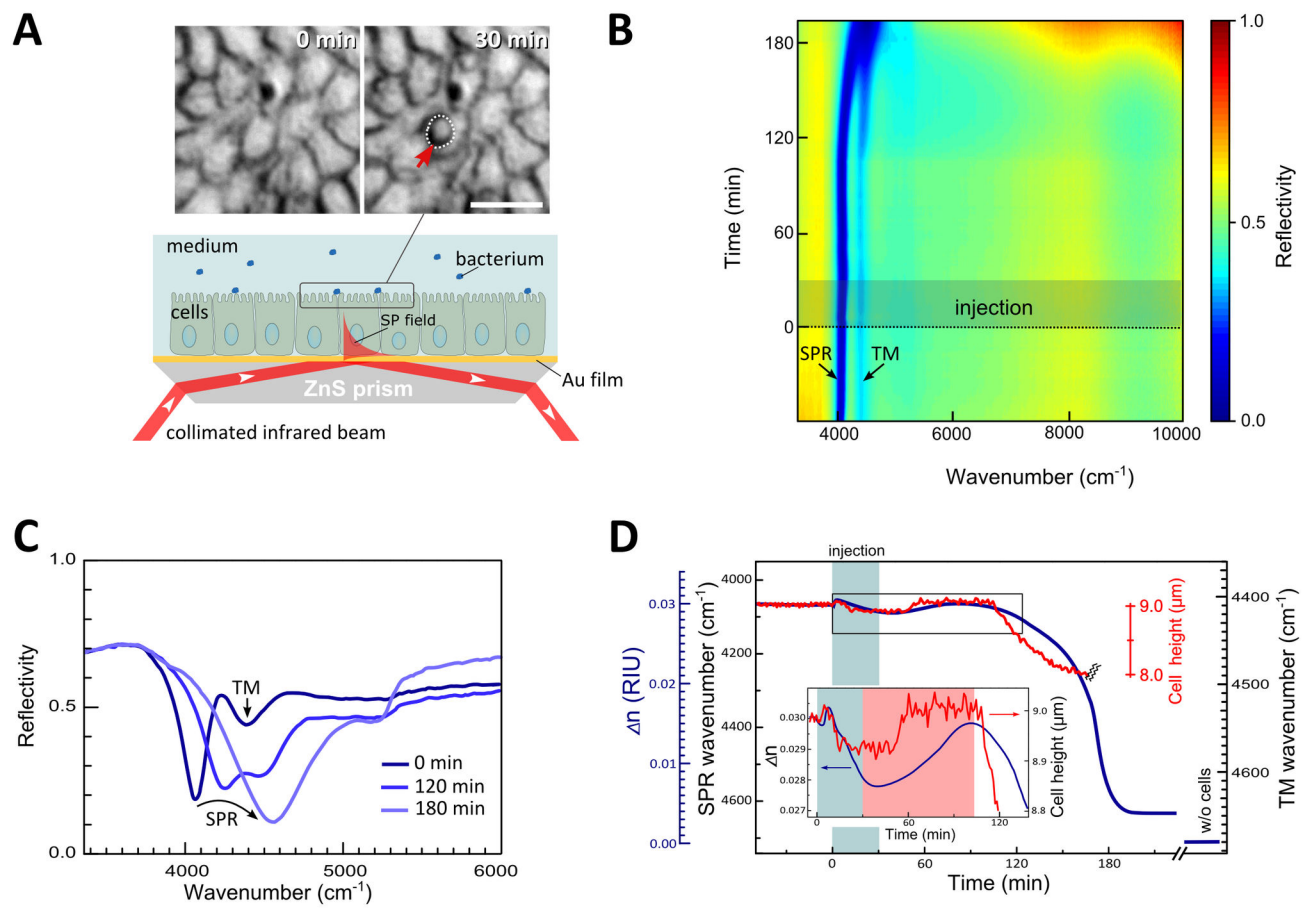
SPR and waveguide mode spectroscopy of infected MDCK cell monolayers. **A**. **Experimental setup**. The lower panel illustrates an MDCK cell layer cultured on a ZnS prism coated with 20 nm Au film, as described in Materials and Methods. The prism with cells was mounted on a flow chamber and exposed to *E*. *coli* strains, which were continuously injected into the flow chamber. Cells and bacteria were imaged simultaneously with the IR-SPR measurements by light microscopy, as we previously described [14]. The upper panel shows images, obtained by microscope, of cells immediately upon (t=0 min) and 30 min after (t=30 min) EPEC-*wt* injection. Encircled are cell-adhering bacterial microcolonies following 30 min of MDCK cell exposure to EPEC. The cell layer was monitored by IR-SPR, in which a collimated and polarized infrared beam from the FTIR spectrometer was reflected from the cell/Au/ZnS prism assembly at an angle corresponding to the surface plasmon (SP) excitation in the Au/cell interface. The typical decay length of the SP field is 2 µm and 50 µm in the vertical and lateral directions, respectively. **B**. **Time evolution of the infrared reflectivity spectrum (R_p-polarized_ /R_s-polarized_) upon host cell-bacteria interaction**. EPEC-*wt* injection was started at t=0 and continued for ~30 min (highlighted by a darkened box). The entire measurement lasted ~ 200 min. Note the gradual broadening and blue-shift of the SPR band and its merging with the TM_01_ waveguide mode. **C**. **Representative infrared reflectivity spectra immediately upon, and after 120 or 180 min of bacterial injection**. Upon bacterial infection the sharp surface plasmon reflectivity dip broadens and becomes blue-shifted. Note the gradual disappearance of the additional reflectivity dip (4400 cm^-1^) corresponding to the TM_01_ waveguide mode propagating in the cell monolayer. **D**. **Time dependence of the SPR wavenumber (main y-axis, left) and the corresponding refractive index of the cell layer *Δn* (secondary blue-colored y-axis, left) during bacterial infection (blue line)**. *Δn* is the difference between the refractive indices of the cell monolayer and the buffer medium *Δn*=*n*_*cell*_-*n*_*medium*_. EPEC-*wt* infection results in a significant blue-shift of the SPR wavenumber, indicating a decrease in *Δn*. The *Δn*=0 at the end of the experiment has been measured after completely removing cells from the substrate by trypsinization. The red line shows the wavenumber of the TM_01_ waveguide resonance in the course of EPEC-*wt* infection. Its change reveals a 1 µm reduction in the average cell monolayer height (secondary right y-axis) (from h=9 µm to h=8 µm) after ~160 min of infection. Thereafter, the TM_01_ resonance becomes smeared and disappears (double zigzag line). This indicates disruption, specifically that the cell monolayer integrity is severely disrupted, and does no longer support the propagation of the waveguide mode. *The* *inset* is a zoom-in of the boxed area in the main plot, essentially showing the *Δn* (t) and cell height dependences recorded for early infection times.

A tight MDCK cell monolayer cultured on a ZnS prism coated with a 20 nm-thick Au film was exposed to EPEC-*wt* infection in a flow chamber of the IR-SPR set-up (**see Materials and Methods and** Figure **1A**). The infrared reflectivity spectra at an oblique incidence were recorded before and after introducing the pathogen. The entire SPR experiment was followed simultaneously by light microscopy (Figure **1A** and Figure **S1**). 

#### Analysis of the surface plasmon resonance (SPR)

The infrared reflectivity dip corresponding to the SPR for cells pre-exposed to bacteria appears at 4080 cm^-1^ (Figure **1B, C** **; t < 0 min**). After ~180 min of EPEC-*wt* infection, the SPR shifts toward 4700 cm^-1^; this value approaches that of a cell-free Au surface. An additional minor reflectivity dip at a larger wavenumber (~4400 cm^-1^) corresponds to the TM_01_ waveguide mode (see Figures **1B, C**). The feature at 5200 cm^-1^ is contributed by water absorption [21], and is therefore not related to surface plasmon. 

The surface plasmon resonance wavenumber yields the effective refractive index of the cell layer, *n*_*cell*_ , see Eq. **1** [13]. We found that for a confluent cell monolayer, *n*_*cell*_=1.29 and for cell medium *n*_*medium*_=1.26 at ν=4000 cm^-1^ [21]. Figure **1D** shows the temporal dynamics of *Δn*=*n*_*cell*_-*n*_*medium*_ during bacterial infection. As indicated previously [14,16,24], *Δn* is most affected by the lower basal-lateral regions of the host cell monolayer because of the ~ 2 μm SPR probing depth [13,16]. Immediately after bacterial injection (at an MOI of 10), *Δn* slightly increases, most likely due to medium exchange. Throughout the injection stage when activated bacteria are introduced into the flow chamber (t=0-30 min), *Δn* gradually decreased to 0.0289. During the subsequent ~60 min, *Δn* recovered, essentially reaching its initial values (Figure **1D** **; see inset**). After approximately 120 min, the *Δn* value dropped sharply and irreversibly for about 60 min, and reached a refractive index value of *Δn*=0.0037 (Figure **1D**), which slightly exceeds that of a stripped-off prism (i.e., a prism without cells) bathed with plain growth medium (i.e. *Δn*=0). It is noteworthy that cell infection at a lower MOI resulted in a slower rate and delayed the drop in host cell colonization and in *Δn*, respectively (Figure **S2**), suggesting that the timing of the *Δn* drop is dependent on host cell infection levels. 

#### Analysis of the TM_01_ waveguide resonance

We have recently shown that the TM_01_ resonance can be used to determine the average height of cells comprising a monolayer, whereas the magnitude of the resonance reflects the cell height homogeneity and intercellular connectivity [23,24]. Here, we tracked the cell height by analyzing the wavenumber of the TM_01_ resonance. Before introducing the bacteria into the flow chamber (i.e., at t<0 min), we obtained an average cell height of 9.0±0.05 µm (Figure **1D**). However, immediately upon infection with EPEC-*wt*, the cell height slightly decreased by 0.12 µm for 35 min (t=10-45 min), and then rapidly recovered to its original value, which remained essentially unchanged for an additional 50 min (t=50-100 min). This minor fluctuation was most probably contributed by the exchange of plain medium with that containing the bacteria. After t=100 min, the cell height decreased by 1 µm over a time span of 60 min (t=100-160 min; Figure **1D**). At later times (i.e., t> 160 min), the TM_01_-waveguide resonance was smeared and eventually disappeared, most likely because of a severe impairment of the cell monolayer integrity. Taken together, these data suggest that the average cell height is a sensitive indicator of early effects that the bacteria have on host cell monolayer structure and integrity, because this feature responded earlier and faster than the refractive index measured by SPR. 

### Temporal profiles of *Δ*n and cell height are type-III secretion-dependent

**Figure 2:**
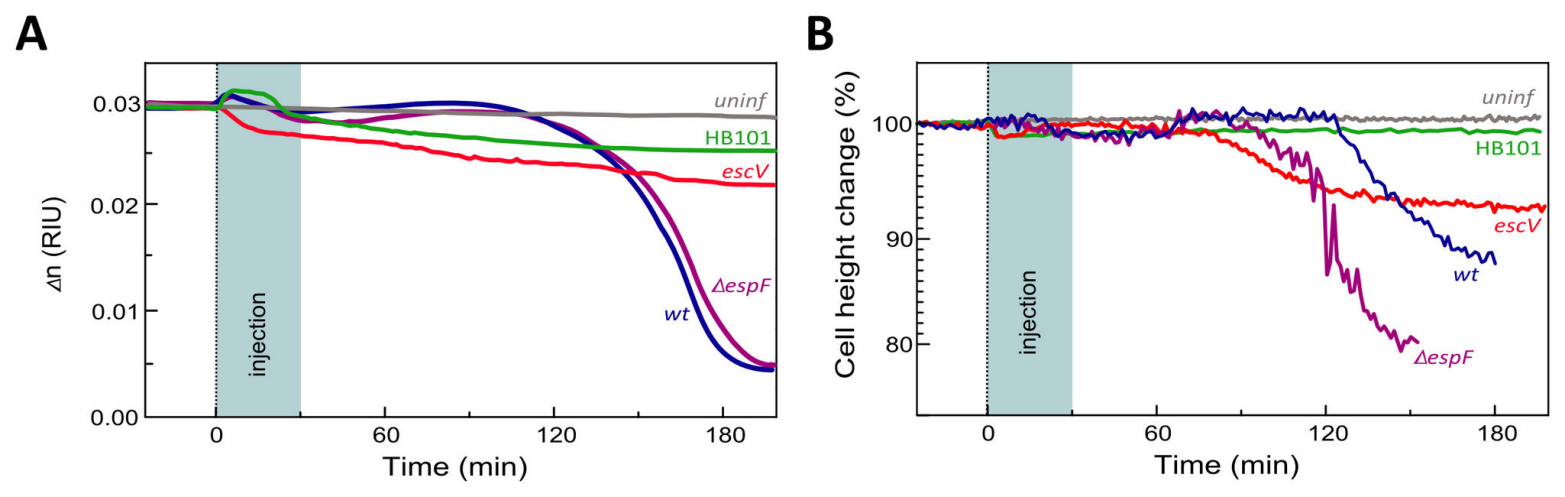
Temporal changes in the MDCK cell monolayer refractive indices and the average cell height. **A**. **Time-dependent changes in the refractive index**. Confluent MDCK cell monolayers that formed after 6-7 days of culturing on an Au-coated prism were exposed to EPEC-*wt* (blue), EPEC-*escV* (red), EPEC-∆*espF* (purple), non-pathogenic HB101 laboratory bacteria (green), or just exposed to bacteria-free growth medium–uninfected (gray). **B**. **Time-dependent changes in the TM_01_ waveguide mode**. The cell height was calculated from the waveguide (TM_01_) resonant wavenumber. Since the initial cell height in these experiments varied from h_0_=8.1 µm to h_0_=11.2 µm, we show the relative cell height normalized to its initial value, i.e., h_0_ is 100%.

Protein effectors translocated into the epithelial host cells affect the cytoskeleton, the intercellular junctions, and the membrane traffic [9]. It is therefore reasonable to hypothesize that these effects would cause structural alterations in the host cell that will be sensed as changes in its refractive index and height. To test this hypothesis, MDCK cell monolayers were infected with either the T3SS-defective EPEC-*escV* or with the non-pathogenic laboratory bacterial strain HB101, which intrinsically lacks T3SS, or the cell monolayers remained uninfected. In the latter case, cells were exposed to plain growth medium containing small amounts of LB, equivalent to those introduced when cells are exposed to bacteria. Figures **2A and 2B** compare the refractive index and cell height dynamics of cells exposed to EPEC-*escV*, HB101, or uninfected cells, to those of EPEC-*wt*-infected cells. 

Figure **2A** shows that the refractive index of uninfected cells remained essentially unaffected over the entire experimental time (approximately 180 min). In all experiments in which cells were exposed to bacteria, a small transient variation of the cell refractive index was observed upon injection. As previously noted, we attribute this to the effects produced upon injection of bacteria-containing medium. Infection with EPEC-*escV* or HB101 resulted in a gradual decrease in the cell refractive index (*Δn*_*escV*_=0.0215, *Δn*_HB101_=0.0259 over 180 min). Nonetheless, the reduction in *Δn* values was much smaller than what was observed after 180 min for EPEC-*wt*-infected cells (*Δn*_*wt*_ =0.0037). These data suggest that translocated type-III-secreted effectors decisively contribute to the unique initial fluctuations and to the significant subsequent reduction in the refractive indices of EPEC-*wt*-infected cells.

Figure **2B** shows that for uninfected cells, the average height of the cell monolayer remains nearly constant over time. A minor drop in the average cell monolayer height (1-2%) was observed in all cases when bacteria were introduced into the flow chamber. In the case of EPEC-*wt*- and EPEC-*escV*-infected cells, however, the average cell height recovered after ~60 min, remained steady for an additional ~ 60 min, and then it sharply dropped. However, unlike EPEC-*wt*-infected cells, where cell height dropped by 10% in the course of 60 min, EPEC-*escV*-infected cells exhibited a slower and smaller reduction in cell height (6%). Interestingly, the experiments with EPEC-*wt*-infected cells always showed that the waveguide mode disappeared approximately 160 min after bacteria injection, whereas with EPEC-*escV* infection, the waveguide mode was still detectable up to 4 hrs post-infection. These results suggest that type III secretion-independent (EPEC-*escV*) mechanisms operate to decrease the host cell monolayer height, but that type III-secreted effectors (EPEC-*wt*) contribute an additional effect. 

### EPEC-wt infection promotes the formation of fluid-phase-filled endocytic vesicles reminiscent of (macro)pinosomes

We next applied time-lapse three-dimensional confocal imaging (i.e., 4D microscopy) and TIRF analyses of living cells to search for potential cellular changes that may contribute to the observed variations in the refractive index.

**Figure 3:**
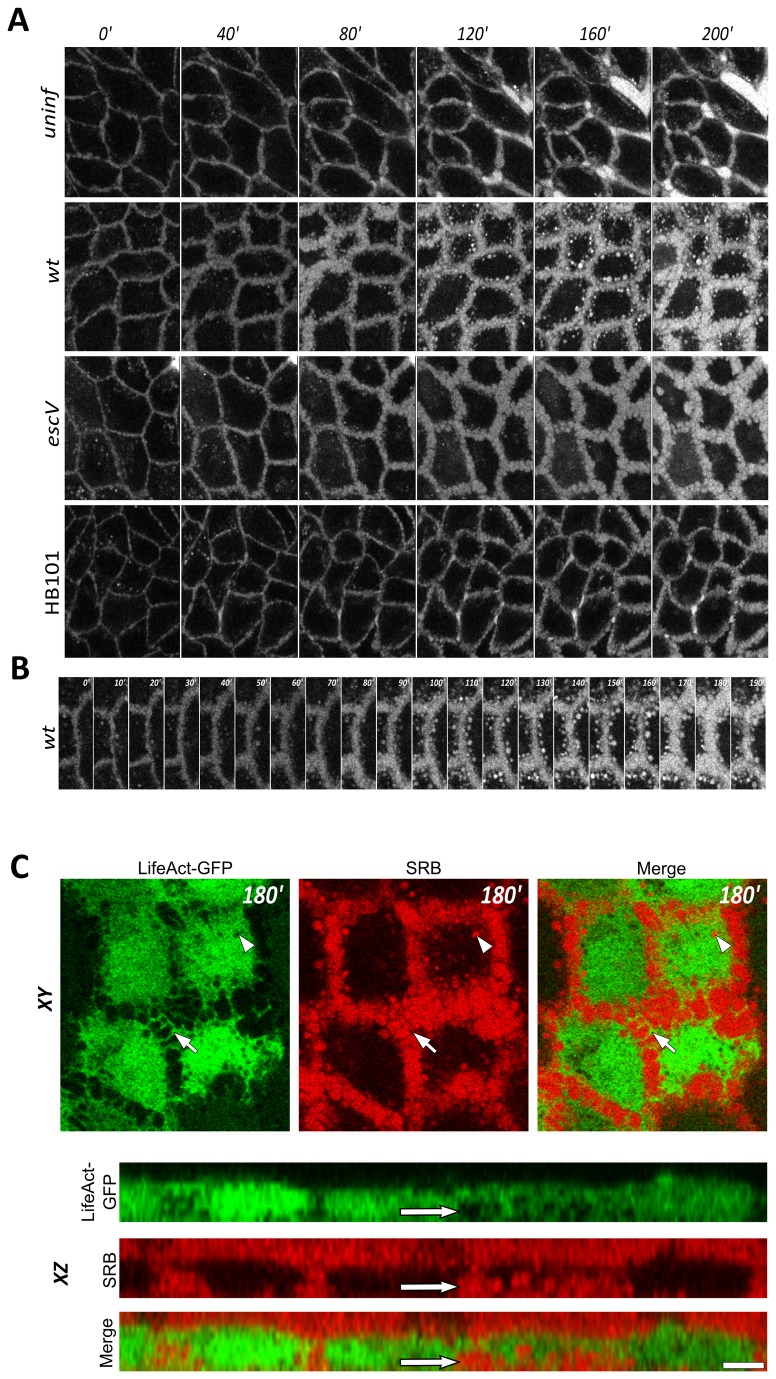
Confocal live cell imaging analysis of MDCK cells expressing LifeAct-GFP exposed to a fluid-phase fluorescent marker, SRB. **A**. **Time series of confocal image sections taken from the lower basolateral regions of an MDCK cell monolayer**. A confluent MDCK cell monolayer, cultured on a multi-well plate (see Materials and Methods), was co-exposed to MEM medium containing the fluorescent fluid-phase marker, SRB (1µM), and the indicated bacterial strains, or to bacteria-free growth medium (uninfected). Note the pronounced enlargement of intercellular spaces in EPEC-*escV*- and EPEC-*wt*-infected cells compared with uninfected cells. These intercellular expanding spaces became visible ~80 min after cell exposure to the microbes. Also note the appearance of SRB-filled vesicles in the cytoplasm of EPEC-*wt-*infected cells. For clarity, the LifeAct-GFP labeling was omitted from the images. Scale bar: 20 µm. **B**. **Zoom of the intercellular interface of EPEC-*wt*-infected cells**. The image series clearly shows the gradual expansion of intercellular spaces filled with SRB and the emerging fluid-phase-filled endocytic vesicles over time. For clarity, the LifeAct-GFP labeling was omitted from the images. **C**. **Visualization of LifeAct-GFP and the extracellular SRB probe at XY and XZ resolutions**. An MDCK cell clone stably expressing LifeAct-GFP (green) was exposed to SRB (red) and EPEC-*wt*-containing medium. A confocal image at XY and XZ resolutions was taken 180 min after EPEC infection. The XY section, which was taken at a lower basal-lateral cell region (~1.5 µm above the cell-substrate interface), shows the actin-labeled tubular-ruffles (green) protruding into the SRB-labeled intercellular spaces (red, indicated by an arrow). Large intracellular SRB-containing vesicles are seen in these cells (indicated by an arrowhead). The XZ section shows a gap between the basal portion of the cell and the underlying substrate, which is filled with the fluid phase SRB marker (indicated by an arrow). Scale bar: 10 µm.

To simultaneously visualize the host cell architecture and its surrounding medium, we used a two-color labeling approach [16]; the MDCK cell volume was labeled by ectopically expressing the F-actin probe, LifeAct-GFP [30], and the extracellular surrounding medium was visualized by adding the hydrophilic dye, SRB [32]. Cells were then exposed in the confocal set-up to HB101, EPEC-*escV*, or EPEC-*wt* in medium containing SRB (Figures **3** and **S3**). Since our SPR experiments mostly sensed basal cellular regions (up to 2 μm above the cell-substrate interface), our initial analyses focused on confocal images taken from the same sections of the host cells. Analysis of the fluid-phase marker (SRB) distribution revealed that the intercellular gaps in these particular regions were considerably enlarged only in cases when cells were exposed to bacteria (Figure **3A**). Actin-based ruffle-tube-like networks of interconnecting cells seemed to protrude from these enlarged areas (Figure **3C** **, confocal XY resolution; indicated by an arrow**). The appearance of dilated intercellular spaces became apparent ~80 min after cell infection with EPEC-*wt* or EPEC-*escV* strains, and about 120 min after cell exposure to HB101. Interestingly, however, large SRB-filled vesicles (up to 1.5 µm in diameter) have only been visualized in the cytoplasm of EPEC-*wt*-infected cells. These vesicles appeared 90 min after infection and their abundance progressively increased over time (Figure **3B**). Notably, the timing of the vesicles' appearance coincided with the onset of the sharp drop in the cell refractive index (see Figure **2A**). The fact that these (macrp)pinsome-like vesicles emerged exclusively in EPEC-*wt*-infected cells suggests that type III-secreted effectors induced their formation.

### EPEC-wt infection induces detachment of the host cell from the underlying substratum

In some cases, we observed an extensive penetration of the SRB marker into the host cell-substrate interface (Figure **3C**, confocal XZ resolution; indicated by an arrow). This event was exclusively induced by EPEC-*wt* infection and was initiated ~90 min after cell exposure to the pathogen. This suggests that EPEC-*wt* infection may cause some degree of host cell dissociation from its underlying substratum. 

**Figure 4:**
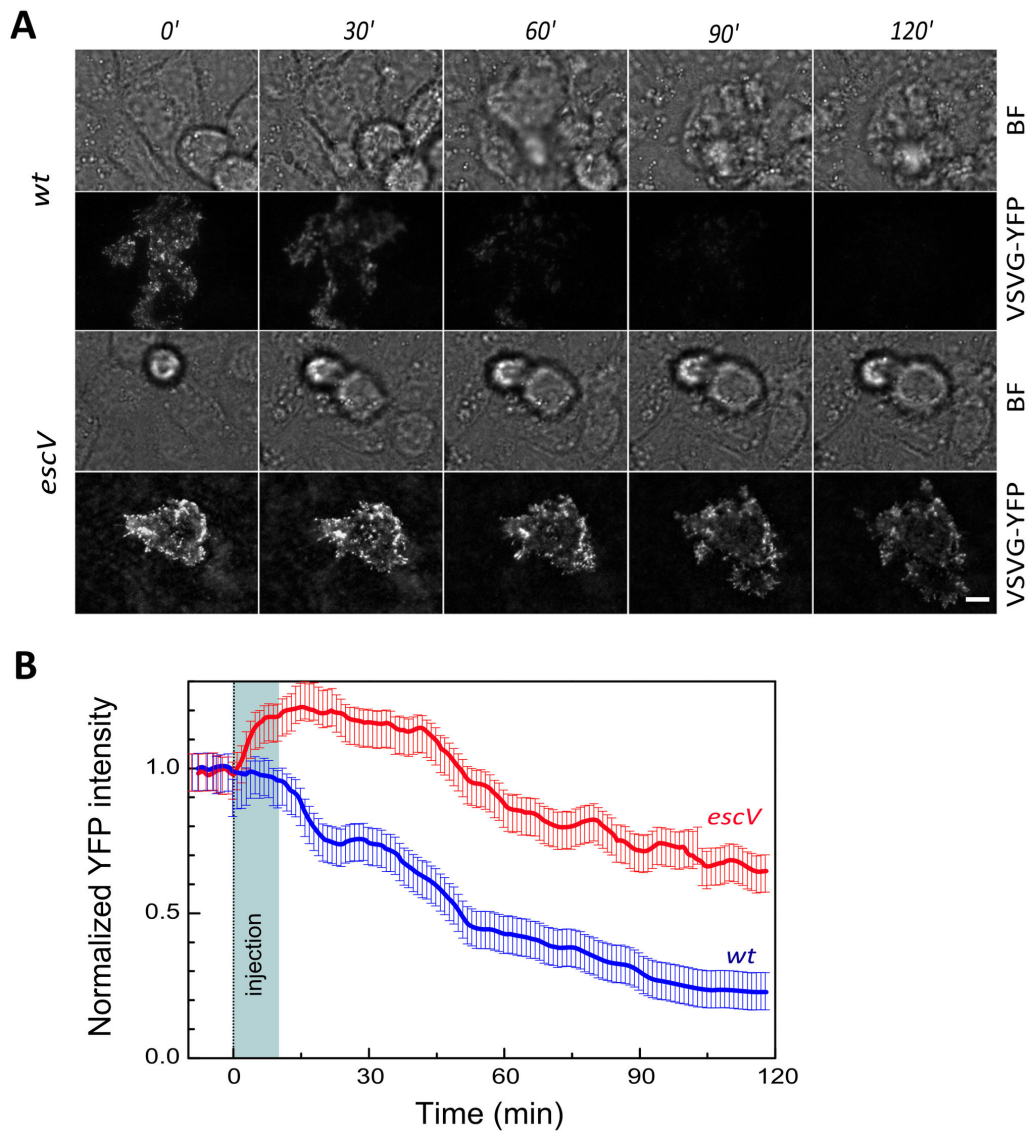
Analysis of MDCK host cell basal membrane attachment to the substrate by TIRF microscopy. Confluent MDCK cell monolayers transiently expressing VSVG-YFP were grown on glass bottom dishes and imaged by TIRF and bright field (BF) microscopy. Images were acquired for at least 20 min prior to the addition of bacteria (not shown), immediately upon cell exposure to EPEC (t=0 min) and at subsequently at 1 min intervals. **A**. **Representative images**. Representative fluorescent and BF images of EPEC-*wt*- and EPEC-*escV*-infected cells are shown at the indicated times. Arrows in the BF images point towards cells that bulged out of the host cell monolayer. About 30% of the cells in the monolayer seemed to show this detaching behavior after 120 min of infection with EPEC-*wt*. In contrast, none of the host cells bulged out and detached from the cell monolayer upon EPEC-*escV* infection. The rounded cells seen in the BF images of the EPEC-*escV*-infected cells are cell-like floaters swept accidentally into the optical field. **B**. **Quantitative analysis**. Data plotted are a moving average of intensity measurement for at least 5 cells corrected for photobleaching and normalized to t=-5 min (pre-infection).

To further address this presumption, we performed TIRF microscopy capable of visualizing at high lateral resolution the cell-substrate adhesion interface (up to 0.1 µm above the cell substrate [33]). In these experiments, VSVG-YFP, which was transiently expressed in MDCK cells, served as a fluorescent marker of the host cell basal plasma membrane [34]. Our TIRF studies showed an immediate and significant reduction over time of the VSVG-YFP fluorescence levels upon cell infection with EPEC-*wt* (Figure **4**). Infection with EPEC-*escV* resulted in a complex change in the fluorescence intensity profile; during the initial ~30 min of cell exposure to the bacteria, the fluorescence intensity increased and then it gradually decreased over time. Nonetheless, the fluorescence levels of VSVG-YFP in the EPEC-*escV*-infected cells always remained considerably higher than those measured for EPEC-*wt-*infected cells (Figure **4**). This suggests that type-III-secreted effectors trigger the removal of the host cell plasma membrane from its underlying substratum, most likely due to detachment. These results are consistent with the localized infiltration of fluid-phase materials into the host-cell substrate interface observed by confocal analyses (Figure **3C**). Interestingly, bright field microscopy revealed that during EPEC-*wt*, but not EPEC-*escV* infection, a small fraction of the host cells (~30%) changed their shape and gradually bulged out (although not completely) of the host cell monolayer (Figure **4A**, indicated by arrows). These results suggest that type III-secreted effectors can break-down the integrity of the host cell monolayer by causing partial host cell detachment from its basal substrate. 

### EspF has no significant impact on EPEC-induced changes in the host cell refractive index, but it affects the cell height

EspF is a protein effector with multiple functions (reviewed in 35). One well-studied function of EspF is its involvement in disrupting the epithelial barrier functions mediated by the tight junctions [36–38]. Bacteria lacking *espF* (EPEC-∆*espF*) are capable of colonizing the host, but have limited ability to disrupt the tight junctions. We hypothesized that these bacteria may also have difficulties in disrupting the host cell monolayer integrity and therefore they decreased its ability to induce water invasion into the basal portions of the host, and consequently decreased the host’s *Δn*. Our results, however, clearly show that not to be the case. MDCK cell infection with EPEC-∆*espF* at ~10 MOI resulted in a time-dependent profile of changes in *Δn* similar to that observed for EPEC-*wt-*infected cells (Figure **2A**). Cell infection with a lower MOI resulted in delayed host cell colonization and a drop in *Δn* (Figure **S2**). Interestingly, however, infection with EPEC-∆*espF* (10 MOI) caused an earlier and stronger decrease in the host cell height than did EPEC-*wt* (Figure **2B**). These results suggest that although EspF does not play a role in decreasing the host cell monolayer refractive index, it may play a role in maintaining its height.

## Discussion

Recently we showed that IR-SPR can be used as a biophysical tool for sensing in real-time and in a label-free manner the processes taking place in epithelial cells [13,14,16]. Moreover, we showed that a tight epithelial monolayer supports the propagation of a waveguide (TM) mode in the infrared wavelength range [23]. This waveguide mode enables highly sensitive real-time monitoring of the cell height and the epithelial cell monolayer integrity [23,24]. Here, we have further utilized our newly developed technology for sensing host cell-pathogen interactions. Specifically, we studied the effects of EPEC infection on an epithelial host cell monolayer. EPEC is an extracellular pathogen that infects the apical cell surface of host epithelial cells [5,7]. Since the IR-SPR methodology senses mainly the lower basolateral portion of the host, the effects we measured herein are those that the microbe transmits from apical infection sites to basolateral regions of the host cell. This conclusion relies on data obtained in the MDCK cellular model. Additional experiments should be done to establish whether this effect also takes place in other polarized epithelial models that are more physiologically relevant as hosts for EPEC infection, e.g., T84 or Caco-2 cell monolayers; see ref [39].

Our data showed that transient and minor variations in the cell refractive index occur during the initial 90 min of cell exposure to EPEC-*wt* (see Figure **1D** **inset**), but not to EPEC-*escV* or the non-pathogenic HB101 strains (Figure **2A**). Hence, we believe that these early effects of the host are contributed by type III-secreted protein effectors. Following ~ 120 min of infection, a robust decrease in the refractive index was observed. Which changes in the host cell have contributed to the sharp reduction in the refractive index? We reasoned that infiltration of a watery environment, particularly into the cellular regions sensed by the IR-SPR, reduces their refractive index. Confocal optical microscopic analyses of MDCK cell monolayers (Figure **3**) yielded results that clearly concurred with this interpretation. For instance, these experiments showed type III-dependent generation of large vesicular-like structures filled with extracellular fluid (SRB) in the lower lateral cytoplasmatic regions of the host cells (Figures **3A&B**). The generation and accumulation of these vesicles have introduced a watery content into the cytoplasm, which contributed, at least partially, to the steep drop in the refractive index of EPEC-*wt*-infected cells (Figures **1D**&**2A**). It is worth noting in this context that epithelial cell infection by the related pathogen enterohemorrhagic *E. coli* has also been reported to induce macropinocytosis [40], which is important for inserting a shiga-like toxin secreted by the microbe into its host. However, the role of EPEC-elicited basolateral (macro)pinocytosis remains to be elucidated.

Interestingly, the intercellular spaces between EPEC-*wt*-infected cells seem to be considerably enlarged and interconnected by a network of ruffled tubular structures (Figure **3C** **, confocal XY resolution**). These morphological changes were not unique to EPEC-*wt-*infected cells, because they were also seen in EPEC-*escV*-infected cells, and to some extent in cells exposed to HB101. It is reasonable to assume that these enlarged basolateral intercellular gaps are filled with a watery environment and therefore contribute to the rather moderate decrease in the refractive indices observed in EPEC-*escV*- and HB101-infected cells (Figure **2A**). 

Both the confocal and TIRF (Figures **3C**&**4**) microscopic analyses showed that infection with EPEC-*wt* or EPEC-*escV* caused a certain degree of host cell detachment from the underlying substratum. However, this effect was far more apparent in EPEC-*wt*-infected cells. Cell detachment from the substrate definitely lead to a watery extracellular medium penetrating into host cell-substrate interfaces; thus, this detachment contributes to the steeper reduction in the refractive indices of EPEC-*wt*-infected cells (Figures **1A**&**2A**). This is consistent with previous reports that showed that EPEC infection induces type III secretion-dependent host cell detachment from the underlying substratum by modifying the focal adhesions [12]. On the other hand, the observation that EPEC-*escV* also induces some degree of host cell detachment is not entirely surprising, since a type III-secreted effector, EspZ, which is not injected by this bacterial strain, has been shown to enhance signaling from β1-integrins and to stabilize focal adhesions [41]. 

Interestingly, in this context, infection with EPEC-*wt* resulted in a robust decrease in the average host cell monolayer height, as assessed by the position of the TM_01_ waveguide mode resonance (Figures **1D**&**2B**). MDCK cells are columnar epithelial cells, and their height is tightly linked to the cortical actin tension [42,43], which is maintained by intercellular TJs anchored to actin cytoskeletal fibers [11]. Thus, perturbation of TJs is expected to lead to a decrease in cell height and disorganization of the epithelial cell monolayer [15,23,24]. Our waveguide analyses showed that, compared with EPEC-*escV*, infection with EPEC-*wt* results in a more robust decrease in cell height (Figure **2B**). This suggests that type III-secreted effectors, which mediate the disruption of TJs and which affect the actin cytoskeleton (e.g., EspF, Map[4–6,8]), may have contributed to the reduction in the host cell monolayer height. Indeed, we found that EspF potentially contributes to cell monolayer height (Figure **2B**). This finding is interesting in light of previous studies linking EspF with intermediate filaments [44] and the involvement of these intermediate filaments in mediating inter-epithelial cell adhesion, epithelial cell architecture [45–47], and possibly height [48]. Although this hypothesis has yet to be further investigated, the results imply that the IR-SPR set-up can be used as a platform for performing a deletion screen and complementation analyses to identify EPEC protein effectors that play a role in decreasing the host cell refractive index and height. Notably, infection with EPEC-*escV* also caused a reduction in cell height, although at a moderate rate and extent compared with EPEC-*wt* (Figure **2B**). This interesting effect suggests that one or more extrinsic (type III secretion independent) bacterial factors contributed to structural changes in the host. This factor could be EspC, an autotransporter protein secreted by EPEC shown previously to be internalized by the host and to affect its actin cytoskeleton [49]. Another possibility is the outer membrane lipopolysaccharide, which has been reported to cause disorganization of TJs in colonic and ductal epithelia [50,51].

**Figure 5:**
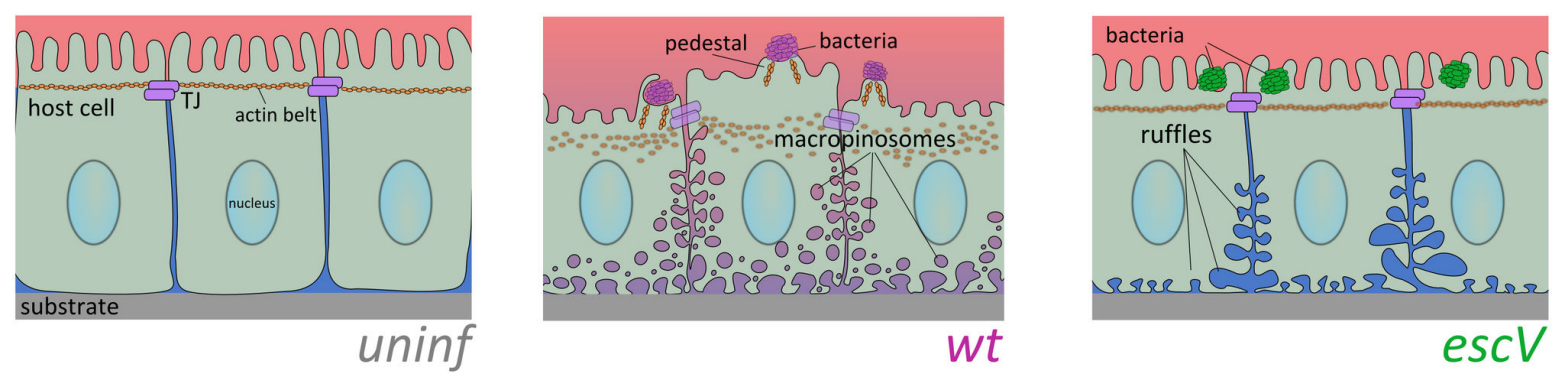
Schematic representation of the effects of EPEC infection on epithelial host cell architecture. In normal uninfected columnar epithelial cells (left panel), the cells maintain close intercellular contacts via tight junctions (TJ), and other junctional complexes (e.g., adherence junctions, gap junctions, and desmosomes). These junction complexes allow the regulation and exchange of different compounds between the underlying tissues and external body cavities, as well as among the connected cells. They are also responsible for maintaining physical contact between the cells and the underlying substrate. In particular, epithelial TJs, which are linked to actin fibers (the apical actin belt), interconnect individual cells into a continuous and rigid epithelial cell sheet. Proper TJ-actin interconnections are essential for developing tall and columnar epithelial cell morphology [42,43]. EPEC-*wt* (middle panel) attached to the apical cell surface of host epithelial cells injects a series of protein effectors into the host cells via T3SS (not shown), among which are effector proteins that subvert the TJs and the actin cytoskeleton, and thereby have broad effects on the epithelial host cell monolayer. Our novel findings suggest that some effector proteins exclusively induce the formation of large fluid-phase-filled endocytic vesicles, reminiscent of (macro)pinosomes. They also evoke substantial host cell detachment from the underlying substratum and a reduction in cell height. The latter could be contributed by a loss of the cortical actin tension maintained by the TJs and the associated cortical actin belt [43]. On the basis of these observations, and combined with other data implying that EPEC disrupts the TJ barrier functions, we can conclude that apically adhered EPEC impair the structural properties of the host in a way that causes a watery extracellular environment to infiltrate into the epithelial sheets, and possibly to the gut’s lumen, which contributes to the diarrheal effect. EPEC-*escV* (right panel) causes host cell basolateral membrane ruffling, expansion of intercellular spaces and some degree of host cell detachment, but does not initiate (macro)pinocytic vesicles formation. EPEC-*escV* has a much weaker effect on the disorganization of the epithelial cell monolayer and the actin cytoskeleton, which still allows the maintenance of the epithelium barrier function. Clearly, these type III secretion independent processes might be contributed by factors associated with the bacterial exterior. One interesting candidate is the EPEC bundle-forming pilus (BFP), which extends out from the bacteria and mediates the initial attachment of EPEC to its host [4]. Following attachment to the host, BFP retracts by a very powerful force generating machinery, bringing to a close apposition the bacterial and host cell surfaces [53]. The host cell may respond to this mechanical force by reorganizing its cortical actin cytoskeleton [54,55], signal transducing proteins [56] and proteins of the junction polarity complex [57]. An intriguing hypothesis is that at least some of these effects have contributed to the type III-independent changes observed upon EPEC-*escV* attachment to its host.

 In summary, we demonstrated here that our newly developed IR-SPR method enables simultaneous detection of multiple and diverse cellular processes affected by EPEC infection. Our overall results suggest the following model (Figure **5**): EPEC-*wt* infection of the apical host cell surface affects at least three major processes in the lower basal-lateral regions of the epithelial barriers: i) enlargement and ruffling of intercellular spaces; ii) induction of (macro)pinocytosis, and iii) disruption of cell monolayer association with the underlying substratum. In addition, our waveguide mode data suggest that EPEC infection elicits a reduction in the epithelial host cell height. All these processes combined may perturb the differentiation state and organization of the epithelial cells in the gut, leading to increased infiltration of watery content into the gut’s tissue, and thereby contribute to the diarrheal effect [52]. The most important advantage of the IR-SPR method is its ability to provide quantitative and simultaneous measurements of the kinetics of various cellular parameters in a large number of cells (~10^5^) with high spatiotemporal resolution, and most importantly, without labeling. Moreover, the waveguide mode enables direct and real-time assessment of the cell height with submicron resolution that hardly could be achieved by optical microscopy. Thus, we foresee that our new development will be further used as a powerful label-free tool for tracking in real-time and with high sensitivity diverse processes of host cell-pathogen interactions*.*

## Supporting Information

Figure S1 **Time-lapse imaging of MDCK cell infection by EPEC.** Cells were visualized by digital zoom light microscopy simultaneously with the SPR measurements. EPEC-wt and EPEC-*escV* microcolonies had been initially observed to associate with the Au-grown host MDCK cells ~30 min after the initial injection of bacteria into the flow chamber (t=30 min). Thereafter, the number of cell-associated microcolonies (indicated by red arrows) increased gradually, reaching maximal levels at t = 60-90 min. Scale bar: 50 µm.
(TIF)

Figure S2 **The effect of multiplicity of infection on the MDCK cell refractive index.** **A**. **Time-dependent changes in the refractive index**. Super confluent MDCK cell monolayers formed after 7 days of culturing on an Au-coated prism were exposed to high (h, ~ 10 MOI) and low (*l*, ~ 5 MOI) doses of EPEC-*wt* or EPEC-*ΔespF*. Time-dependent changes in *Δn* were measured as in Figure 1. **B**. The kinetics of host cell monolayer colonization upon infection with EPEC at different MOIs. Host cell-associated EPEC microcolonies have been visualized as in Figure S1. Optical images of infected cells acquired every 1 min have been processed. Cell-associated bacterial microcolonies were manually counted in an image area of ~0.2 mm^2^, using the ImageJ "Cell Counter" plug-in.
(TIF)

Figure S3 **Time-lapse confocal imaging of an EPEC-infected MDCK cell monolayer.** A super-confluent MDCK monolayer was exposed to EPEC-*wt* and EPEC-*escV* infection in the confocal set-up, as in Figure 3. Bacterial microcolonies appear as dark grape-like shapes in the background of SRB-labeled medium (indicated by red arrows). Similar to the SPR experiments, bacterial microcolonies attached to host cells appeared ~30 min after they were introduced into the flow chamber, reaching maximal levels ~60 min thereafter. Scale bar: 20 µm.
(TIF)

Table S1 **List of bacterial strains.**
(TIF)
