# Supplementary figures and images for: Real-Time Sensing of Enteropathogenic E. coli-Induced Effects on Epithelial Host Cell Height, Cell-Substrate Interactions, and Endocytic Processes by Infrared Surface Plasmon Spectroscopy
Source: PLoS One. 2013 Oct 23;8(10):e78431. doi: 10.1371/journal.pone.0078431 (PMC3806826; doi:10.1371/journal.pone.0078431)

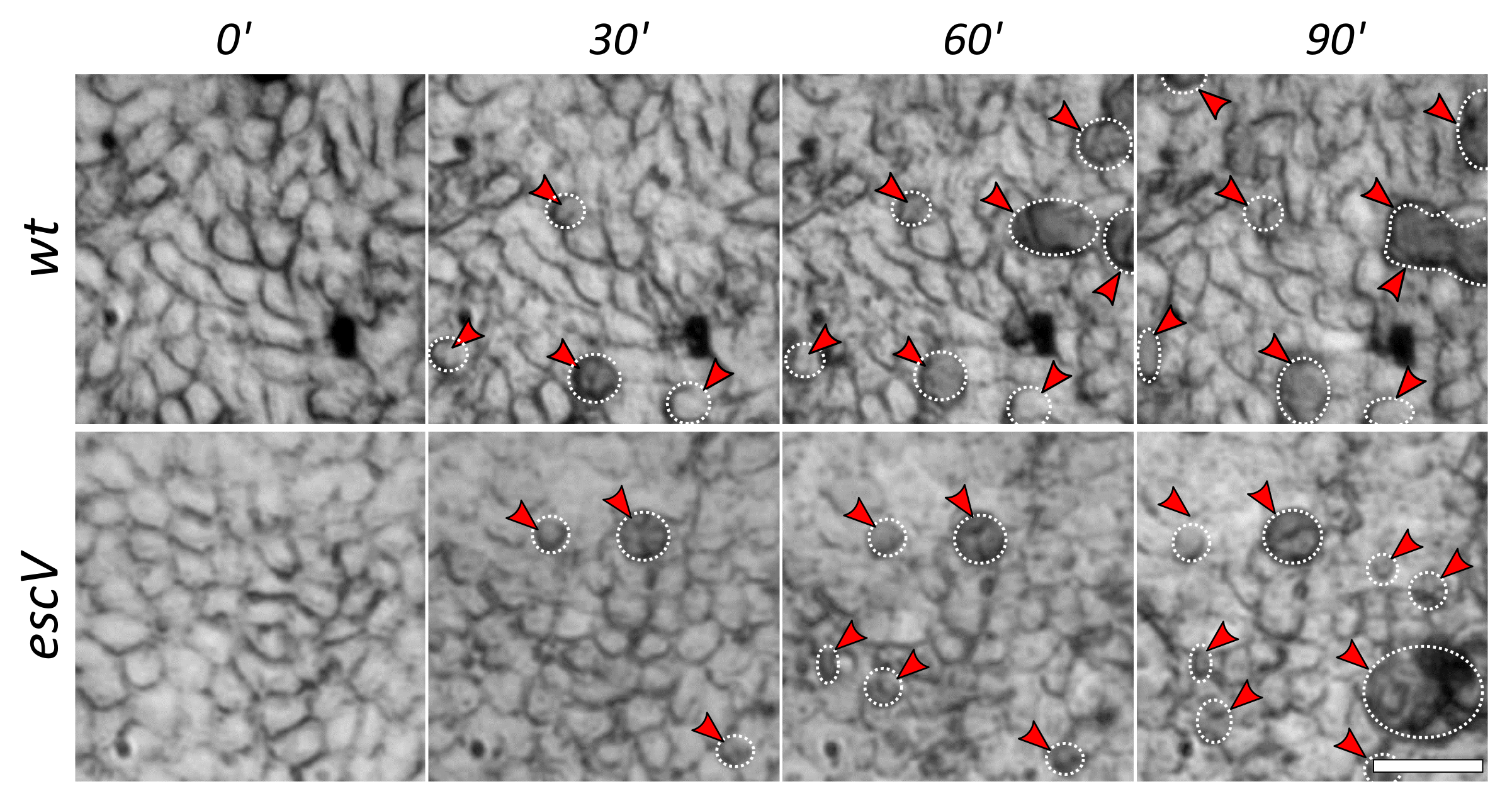

Supplement: Figure S1 — Time-lapse imaging of MDCK cell infection by EPEC. Cells were visualized by digital zoom light microscopy simultaneously with the SPR measurements. EPEC-wt and EPEC-escV microcolonies had been initially observed to associate with the Au-grown host MDCK cells ~30 min after the initial injection of bacteria into the flow chamber (t=30 min). Thereafter, the number of cell-associated microcolonies (indicated by red arrows) increased gradually, reaching maximal levels at t = 60-90 min. Scale bar: 50 µm. (TIF) [file pone.0078431.s001.tif]

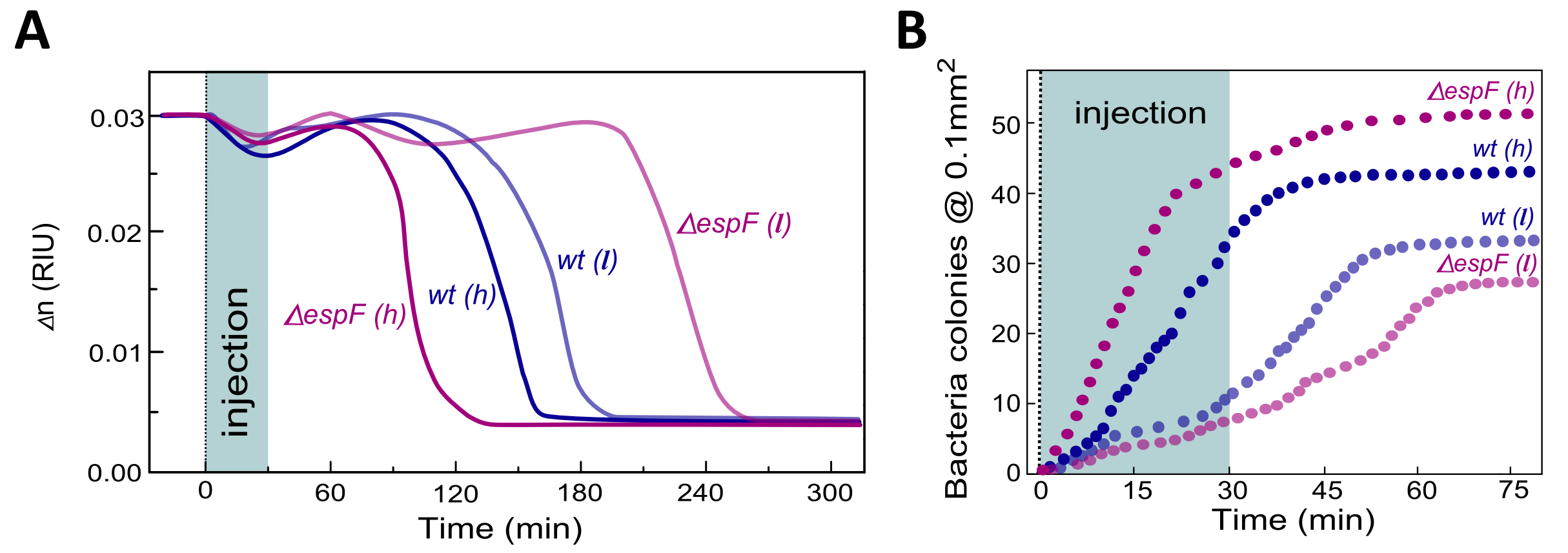

Supplement: Figure S2 — The effect of multiplicity of infection on the MDCK cell refractive index. A. Time-dependent changes in the refractive index. Super confluent MDCK cell monolayers formed after 7 days of culturing on an Au-coated prism were exposed to high (h, ~ 10 MOI) and low (l, ~ 5 MOI) doses of EPEC-wt or EPEC-ΔespF. Time-dependent changes in Δn were measured as in Figure 1. B. The kinetics of host cell monolayer colonization upon infection with EPEC at different MOIs. Host cell-associated EPEC microcolonies have been visualized as in Figure S1. Optical images of infected cells acquired every 1 min have been processed. Cell-associated bacterial microcolonies were manually counted in an image area of ~0.2 mm2, using the ImageJ "Cell Counter" plug-in. (TIF) [file pone.0078431.s002.tif]

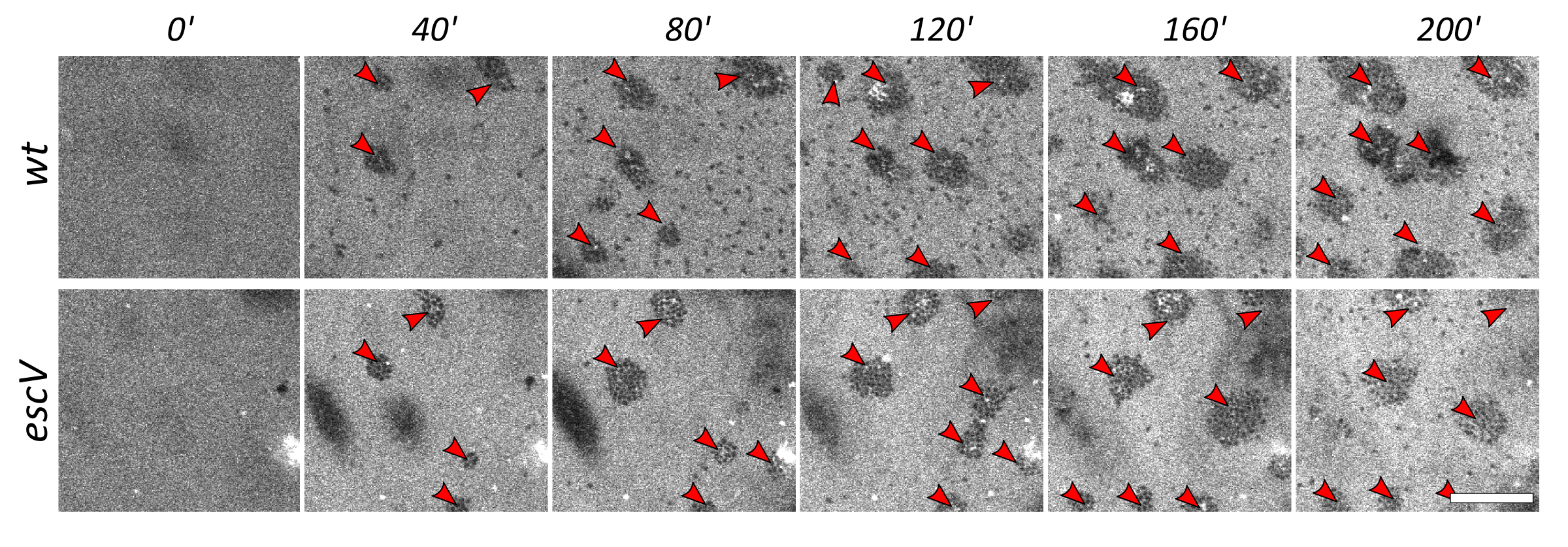

Supplement: Figure S3 — Time-lapse confocal imaging of an EPEC-infected MDCK cell monolayer. A super-confluent MDCK monolayer was exposed to EPEC-wt and EPEC-escV infection in the confocal set-up, as in Figure 3. Bacterial microcolonies appear as dark grape-like shapes in the background of SRB-labeled medium (indicated by red arrows). Similar to the SPR experiments, bacterial microcolonies attached to host cells appeared ~30 min after they were introduced into the flow chamber, reaching maximal levels ~60 min thereafter. Scale bar: 20 µm. (TIF) [file pone.0078431.s003.tif]

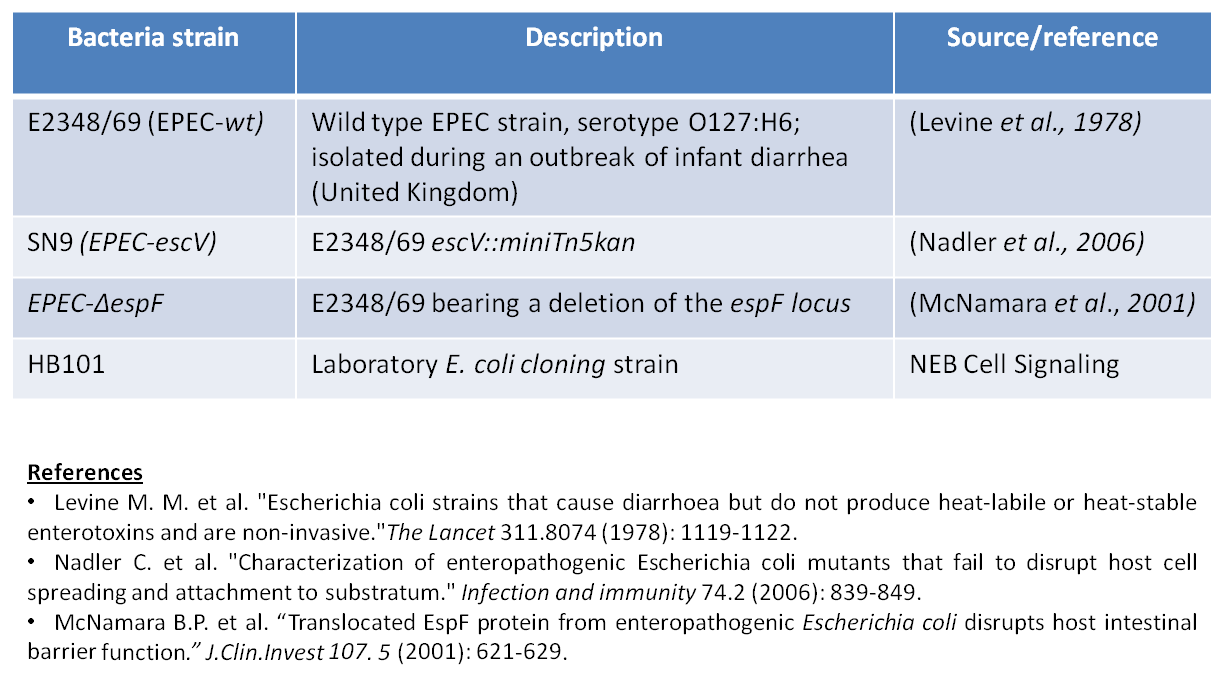

Supplement: Table S1 — List of bacterial strains. (TIF) [file pone.0078431.s004.tif]
